# Treatment first, transport second: rethinking stroke management with mobile stroke units—A systematic review and meta-analysis of controlled trials

**DOI:** 10.3389/fneur.2026.1852802

**Published:** 2026-09-03

**Authors:** Aishah Ibrahim Albakr, Ziyad Masoud Alqahtani, Abdulmonem Mohammed AlSharidah, Adel Abdalla Zeidan, Ahmed Abdulrahman Al Ibrahim, Mohammed Abdulmonem Al Zaher, Khurshid Khan

**Affiliations:** 1 Department of Neurology, Imam Abdulrahman Bin Faisal University, Dammam, Saudi Arabia; 2 Department of Neurology, King Fahad Specialist Hospital, Dammam, Saudi Arabia; 3 Department of Neurology, College of Medicine, Imam Abdulrahman Bin Faisal University, King Fahad Hospital of the University, Dammam, Saudi Arabia; 4 Imam Abdulrahman bin Faisal University, Dammam, Saudi Arabia; 5 College of Medicine and Surgery, Imam Abdulrahman Bin Faisal University, King Fahd Hospital of the University, Dammam, Saudi Arabia; 6 Division of Neurology, Department of Medicine, University of Alberta, Edmonton, AB, Canada

**Keywords:** alteplase, functional outcome, mobile stroke unit, stroke, thrombolysis

## Abstract

**Background:**

Stroke is a significant cause of morbidity and mortality, with profound medical and economic consequences. Accurate diagnosis and minimizing delays in acute stroke management has become a global priority. Mobile stroke units (MSUs) have the potential to effectively shorten key time intervals.

**Objective:**

We conducted this review aiming to investigate the benefits of MSU implementation on functional, safety and time metrics outcomes in acute stroke care. Moreover, we assessed whether the presence of an onboard neurologist is essential for MSU implementation.

**Results:**

The analysis consisted of 10 studies involving 13,110 patients (4,350 MSU; 8,760 EMS). The pooled median difference favors MSU intervention over the control by 26.3 min [95% CI (20.1–32.4), *p* < 0.001], this reduction was statistically significant. The test for subgroup differences between onboard neurologist and remote neurologist configurations was not significant (χ^2^=0.12, df = 1, *p* = 0.7241). Both safety outcomes showed non-significant pooled odds ratios, ICH 1.019 [95% CI (0.71,1.45), *p* = 0.918] and mortality 1.23 [95% CI (0.85, 1.77), *p* = 0.271].

**Conclusions:**

In this review, it was demonstrated that MSUs have markedly improved some of the critical time metrics of acute stroke care through clinically meaningful reductions of ~10–35 min, while maintaining safety outcomes comparable to traditional EMS care. We found no significant differences considering the impact of onboard and remote neurologist models in MSUs, highlighting that remote neurologists in MSUs could potentially be a more cost-effective model.

**Systematic review registration:**

PROSPERO; registration number: CRD420261297860.

## Introduction

Stroke is a significant cause of morbidity and mortality, with profound medical and economic consequences (1). Neuronal loss occurs rapidly and irreversibly, making immediate treatment essential, as stroke management depends on a narrow therapeutic window commonly known as “time is brain” (2). Early administration of thrombolytic therapy is contingent upon fast, accurate diagnosis and minimizing delays in acute stroke management. Consequently, it has become a global priority to enhance patient outcomes and lower secondary complications (2–4). Considering the prognostic factors in stroke, the time to reperfusion therapy represents the most significant determinant of patient outcomes. Evidence indicates that mobile stroke units (MSUs) can effectively shorten this vital timeframe (5).

Conventional emergency medical services (EMS) have the potential to identify suspected stroke and pre-notify receiving hospitals, but they lack on-scene neuroimaging and definitive treatment capabilities. MSUs are equipped with advanced devices and services since it combines the conventional emergency services along with telemedicine technologies, a CT scanner, and specialized trained personnel to decide whether to give thrombolysis or not directly at the scene. By equipping physicians in the prehospital setting with the necessary information and resources to accurately assess patients for the eligibility to administer thrombolysis on-site, this approach can significantly reduce the time from symptom onset to treatment (6).

Emerging evidence suggests that MSUs facilitate faster treatment initiation and potentially improve clinical outcomes (7–9). However, considerable uncertainty remains due to diversity in trial designs and inconsistencies in the definitions of key time intervals. Additionally, earlier studies were often constrained by small study numbers and a lack of comprehensive evaluation across all outcomes, including functional recovery, time efficiency, and safety profiles. Recently, published randomized controlled trials (RCTs) and non-randomized studies point out the need for an updated systematic synthesis. A critical unresolved issue is whether an onboard neurologist is necessary for the operation of MSUs, as this may impact both the overall effectiveness and safety of the service.

This systematic review and meta-analysis of controlled trials aims to offer clinicians, researchers, and policymakers with high-quality evidence regarding the effectiveness, safety and time-sensitive benefits of MSU implementation. By systematically comparing patients' outcomes with standard EMS management in acute stroke care, this study seeks to clarify the clinical and operational value of MSUs and inform evidence-based strategies for their broader adoption.

## Methods

### Study design and registration

The study was conducted as a systematic review and meta-analysis of randomized and non-randomized clinical trials in accordance with the Preferred Reporting Items for Systematic Reviews and Meta-Analyses (PRISMA) 2020 guidelines (10). The review protocol was prospectively registered in the International Prospective Register of Systematic Reviews (PROSPERO); registration number: CRD420261297860.

### Literature search

A comprehensive literature search was conducted in PubMed, Cochrane Library, Web of Science, Scopus, and MEDLINE. Additionally Gray literature was searched via ClinicalTrials.gov and Reference checking, and the final search was completed in July 2025.

The search strategy was developed based on the research objectives and inclusion criteria. a combination of text words, boolean operators, and Medical Subject Headings (MeSH) terms were used and tailored individually to each database. The search was restricted to English-language studies and included both randomized and non-randomized controlled trials, with no timeframe limitations. Full details of the search strategy and database-specific search strings are provided in the Supplementary Data.

All records were imported into the reference management software Zotero, where duplicates were automatically removed. Two reviewers independently screened the titles and abstracts, followed by a full-text review Phase. Disagreements between the two reviewers were resolved through a third reviewer.

### Inclusion and exclusion criteria

Studies were included according to predefined criteria, focusing on patients with suspected acute stroke who received management via MSUs compared with EMS. Both randomized and non-randomized clinical trials published in English were eligible for inclusion. Studies which applied an alternative intervention, comparison group, and non-trial study designs were excluded from the review. Furthermore, included studies were required to report at least one of the predefined main outcomes in order to be eligible.

### Data extraction and main outcomes

The data was extracted through two reviewers independently using a predesigned spreadsheet, and a third reviewer verified all extracted information to ensure accuracy. Any discrepancies were resolved through discussion until consensus was reached.

Extracted variables included study identification details, study characteristics, participant characteristics, and main outcomes of interest. The main outcomes included time metrics, functional outcomes, and safety outcomes. Time metrics encompassed onset-to-thrombolysis time, alarm-to-thrombolysis time, door-to-thrombolysis time, door-to-thrombectomy time, and alarm-to-CT time. Functional outcomes were assessed by the modified Rankin Scale, which measures post-stroke functional independence. Intracranial hemorrhage (ICH) and mortality were used to assess for safety.

### Quality assessment

The methodological quality of the included studies was assessed independently by two reviewers. Discrepancies were resolved through discussion and consensus. The tools used for quality appraisal were the Cochrane Risk of Bias 2 (RoB 2) tool for randomized controlled trials and the Risk of Bias in Non-randomized Studies of Interventions (ROBINS-I) tool for non-randomized studies (11, 12). Each domain within these tools was evaluated independently. The RoB 2 tool assesses five domains of potential bias: (1) the randomization process, (2) deviations from intended interventions, (3) missing outcome data, (4) measurement of outcomes, and (5) selection of reported results. The ROBINS-I tool evaluates seven domains: (1) confounding, (2) selection of participants, (3) classification of interventions, (4) deviations from intended interventions, (5) missing data, (6) measurement of outcomes, and (7) selection of reported results. Each study received an overall risk of bias judgment categorized as low, moderate (some concerns), or high risk of bias, according to the criteria specified in the respective assessment tools.

### Statistical analysis

Most continuous outcomes of interest in the literature are reported as medians with interquartile ranges (IQRs). This is often derived from non-parametric tests and skewed data distribution. Conventional meta-analytic methods typically cannot be directly employed with medians and interquartile ranges unless they are transformed into means and standard deviations. However, most statistical methods used for estimating means typically require the assumption of data normality. Therefore, we analyzed continuous outcomes using the median-based method described by McGrath et al. (13). McGrath approach avoids normality assumption and allows pooling of effect estimates by medians and interquartile ranges (IQRs) without means estimation. The effect estimates (difference of medians) and corresponding variance were estimated by quantile estimation (QE) method. Then the extracted differences of medians were pooled by a random-effect model with DerSimonian–Laird (DL) estimator and 95% confidence intervals for between-study variance. The random effect approach was adopted in continuous and binary outcomes because variability in populations, protocols, and definitions across included studies were expected. Across all outcomes, positive values indicate shorter times or better outcomes favoring MSU. In order to explore the heterogeneity and neurologist involvement role, Subgroup analyses were prespecified by study design (randomized vs. non-randomized) and by neurologist involvement (onboard vs. remote). Random-effects meta-analyses were performed in all subgroups, and between-subgroup differences were assessed using the Q-test for subgroup differences. Studies lacking subgroups of interest or with insufficient number of studies were excluded from that specific subgroup analysis. Binary safety outcomes were reported using odds ratios (ORs) with 95% confidence intervals (CIs). ORs with values >1 indicate fewer adverse events with the MSU. The Mantel-Haenszel method was implemented to pool effect estimates, while the DerSimonian-Laird estimator was applied to account for between-study variance. Heterogeneity was assessed using τ^2^ and *I*^2^ statistics. Heterogeneity was classified as moderate (*I*^2^ of 30% or more), moderate (*I*^2^ of 50% or more), or high (*I*^2^ of 75% or more). Additionally, Sensitivity analyses were conducted using leave-one-out and influence analyses to assess the pooled results. Publication bias was assessed visually by funnel plots. All statistical analyses were performed using R software (version 4.4.0). For reproducibility purposes, full statistical analysis R code is available upon reasonable requests.

## Results

### Study selection and characteristics results

**Figure 1:**
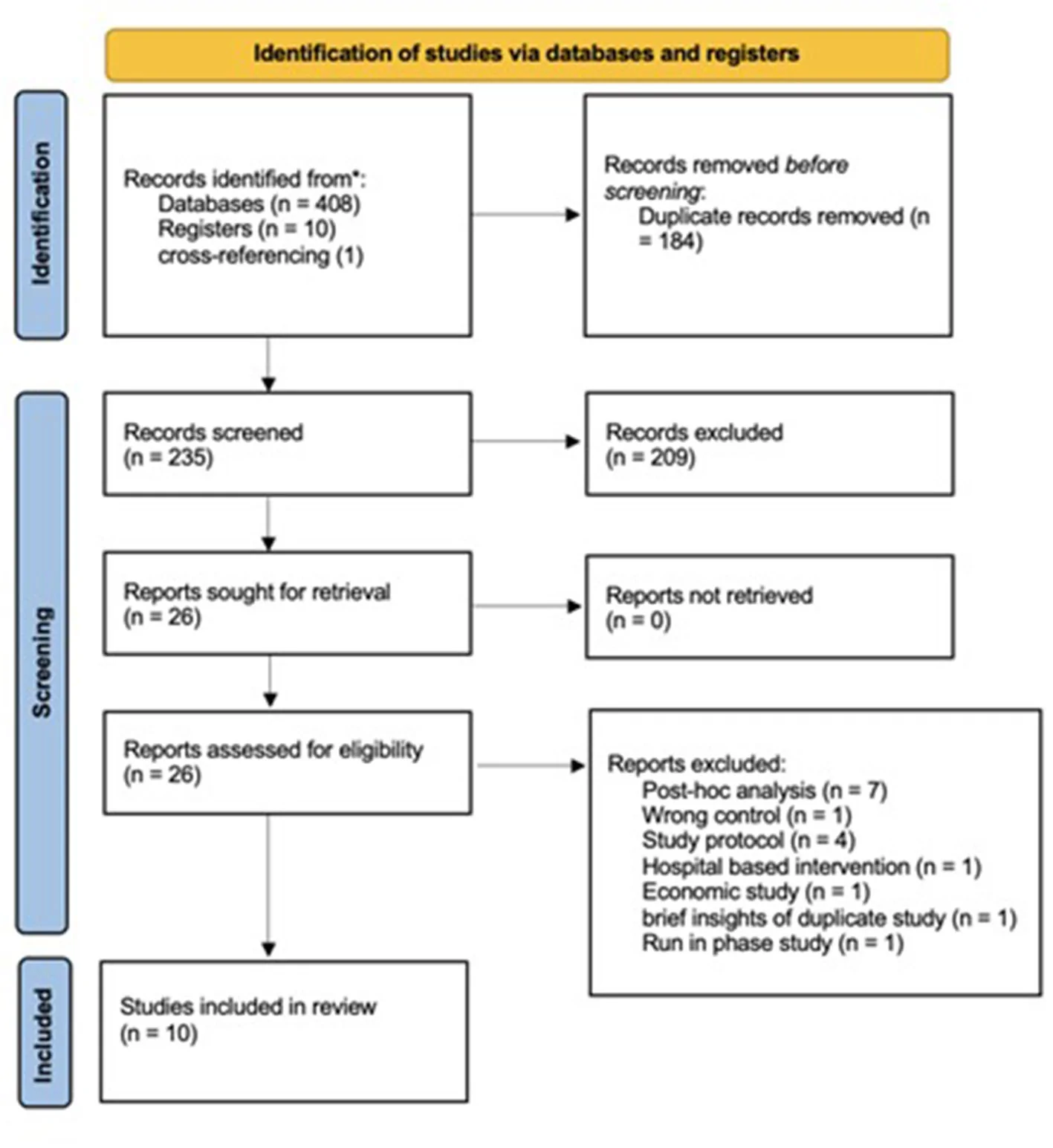
PRISMA 2020 flow diagram for new systematic reviews which included searches of databases and registers only. *Databases and registries include PubMed, Cochrane Central Register of Controlled Trials (CENTRAL), Web of Science Core Collection, SCOPUS, MEDLINE. One study (PHANTOM-S) was identified by cross-referencing.

A total number of 408 records across all databases were identified. 184 were duplicates, 234 were screened by abstract and title, of which 25 full texts were screened to assess for their eligibility, in total, 10 studies were included in the analysis (see Figure 1, for PRISMA diagram).

The analysis consisted of 10 studies involving 13,110 patients (4,350 MSU; 8,760 EMS). Out of these 4 are randomized controlled trials while the remaining are non-randomized controlled trials. All these trials were conducted in 5 countries, most of which were in Germany, China, and Norway. Six studies were conducted in urban settings, three in nonurban areas, and two in mixed settings (see Supplementary appendix 6 for details). The scope was on patients that are suspected of acute ischemic stroke, and some studies specified a subtype such as large-vessel occlusion and eligible cases for thrombolysis. Only two studies reported stroke mimics rates: Helwig et al. (14) 9.5% (MSU arm) versus 3.8% (EMS arm), and Grotta et al. (15) 13.3% (MSU arm) versus 11.4% (EMS arm). The intervention was made by MSUs equipped with CT scanner and telemedicine capabilities. In 5 studies, a neurologist was present onboard, while in the remaining studies, the patient was evaluated remotely by a neurologist (tele-medicine). The controls received medical care via standard EMS. The outcomes that were analyzed such as door to thrombectomy time, alarm to treatment, modified Rankin scale (mRs), safety outcomes (intracerebral hemorrhage, mortality) demonstrated variation in reporting across studies. Detailed characteristics of the included studies are provided in (Table 1). In total ten studies were included in the quality assessment and the risk of bias assessment. Four were rated as having a high risk of bias, and six were at a moderate risk of bias. The detailed version of the risk of bias evaluation and sensitivity analyses are summarized in the Supplementary materials.

**Table 1:** Baseline characteristics of all included studies.

Name of the study	Location	Design	MSU configuration	Number of participants	Age
Helwig et al. (14)	Saarland, Germany	Randomized clinical trial	Onboard neurologist, CT, POC lab, and thrombolysis	Total 116	
				MSU 63	MSU 75 ± 11
				EMS 53	EMS 74 ± 11
Zheng et al. (24)	Ya'an, China	Randomized clinical trial	Remote neurologist. Onboard CT, POC lab, and thrombolysis	Total 207	
				MSU 101	MSU 70.1 ± 11.7
				EMS 106	EMS 70.7 ± 10.3
Larsen et al. (27)	Østfold county, Norway	Non–randomized clinical trial	Remote neurologist. Onboard CT, POC lab, and Thrombolysis	Total 440	
				MSU 166	MSU 72 (57–83)
				EMS 274	EMS 72 (60–81)
Grotta et al. (15)	Houston, USA	Non–randomized clinical trial	Remote neurologist. Onboard CT, POC lab, and thrombolysis	Total 1,047	
				MSU 617	MSU 67 (57–79)
				EMS 430	EMS 65 (55–78)
Nilanont et al. (30)	Bangkok, Thailand	Non–randomized clinical trial	Remote neurologist. Onboard CT, POC lab, and thrombolysis	Total 978	
				MSU 243	MSU 66 (54, 75)
				EMS 214	EMS 67 (56, 77)
Walter et al. (16)	Homburg, Germany	Randomized clinical trial	Onboard neurologist, CT, POC lab, and thrombolysis	Total 100	MSU 72 (59–76)
				MSU 53	EMS 71 (55–75)
				EMS 47	
Zhao et al. (26)	Melbourne, Australia	Non–randomized clinical trial	Onboard neurologist, CT, POC lab, and thrombolysis	Total 2?348	74 (66–83)
				MSU 1,179	
				EMS 136	
Ebinger et al. (31)	Berlin, Germany	Non–randomized clinical trial	Onboard neurologist, CT, POC lab, and thrombolysis	Total 1,543	74 years (±13)
				MSU 749	
				EMS 794	
Liu et al. (32)	China	Non–randomized clinical trial	Remote neurologist. Onboard CT, POC lab, and thrombolysis	Total 149	
				MSU 56	MSU 67.57 ± 9.53
				EMS 93	EMS 64.55 ± 9.70
Ebinger et al. (9)	Berlin, Germany	Randomized clinical trial	Onboard neurologist, CT, POC lab, and thrombolysis	Total 6,182	
				MSU 1,804	MSU 73.9 ± 15.0
				EMS 2,969	EMS 74.3 ± 14.9

MSU, mobile stroke unit; EMS, emergency medical service; POC lab, point-of-care laboratory; CT, computed tomography.

### Quantitative synthesis

#### Time metrics outcomes

**Figure 2:**
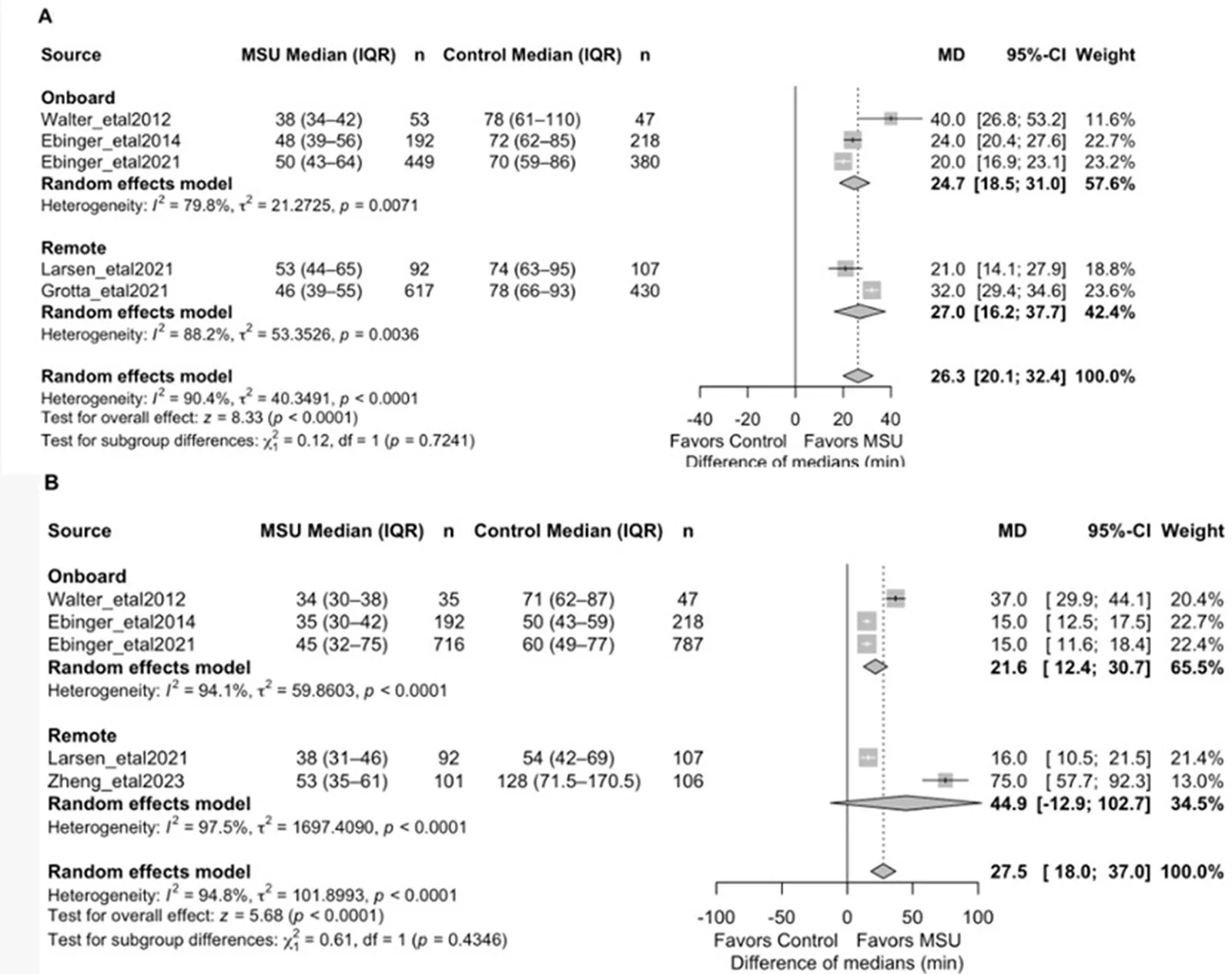
Pooled Medians Difference of Time metrics outcomes. **(A)**: Alarm to thrombolysis time. **(B)**: Time to end of CT.

Five studies (*n* = 1,403 MSU; 1,182 EMS) were included in Alarm to thrombolysis time analysis. The pooled median difference favors MSU intervention over the control by 26.3 min [95% CI (20.1–32.4), *p* < 0.001], this reduction was statistically significant. Three of these studies deployed an onboard neurologist which showed a pooled median difference of 24.7 min [95% CI (18.5, 31.0), *p* < 0.001; *I*^2^=80%]. In addition, the two-remaining studies in the analysis deployed a remote neurologist which achieved similar outcomes with pooled diffenrence of 27 min [95% CI (16.2, 37.7), *p* < 0.001; I^2^ = 90%]. The test for subgroup differences was not significant (χ^2^=0.12, df = 1, *p* = 0.7241) (Figure 2A). Similarly, regarding the onset to thrombolysis time, the analysis involved five studies (*n* = 887 MSU; *n* = 1,029 EMS), which demonstrated a significant reduction of 35.3 min favoring the intervention over the standard medical care, with confidence interval of [16.1, 54.4] (*p* < 0.001; I^2^ = 90%). For the door to thrombolysis time, three studies were analyzed and the pooled analysis showed that MSU was associated with a median reduction of 9.5 min [95% CI (5.9–13.4), *p* < 0.001; *I*^2^= 70%] compared to the EMS control. Across three studies (*n* = 689 MSU; *n* = 543 EMS), the intervention group demonstrated a pooled reduction of 16.5 min in door to thrombectomy time in comparison with the control group [95% CI (12.4,20.7), *p* < 0.001]. This improvement was statistically significant and revealed no heterogenicity across studies (*I*^2^= 0.0%). Five studies (*n* = 1,136 MSU; *n* = 1,265 EMS) were included in the analysis of measuring the time to completion of CT. Implementation of MSU demonstrated a reduction of time to completion of CT with a pooled mean difference of 27.5 min [95% CI (18.0, 37.0), *p* < 0.001, I^2^=90%] favoring the MSU intervention over standard EMS (Figure 2B). In addition, three of the studies were equipped with an onboard neurologist and two studies used a remote neurologist. Both subgroups demonstrated a reduction in completion of CT time, remote neurologist showed higher reduction in time 44.9 min, but was statistically insignificant [95% CI (-12.9,102.7), *p* = 0.128; *I*^2^ = 100%]. The test for subgroup differences was not significant (χ^2^ = 0.61, df = 1, *p* = 0.43). For further details, refer to (Figure 1).

#### Functional outcomes

**Figure 3:**
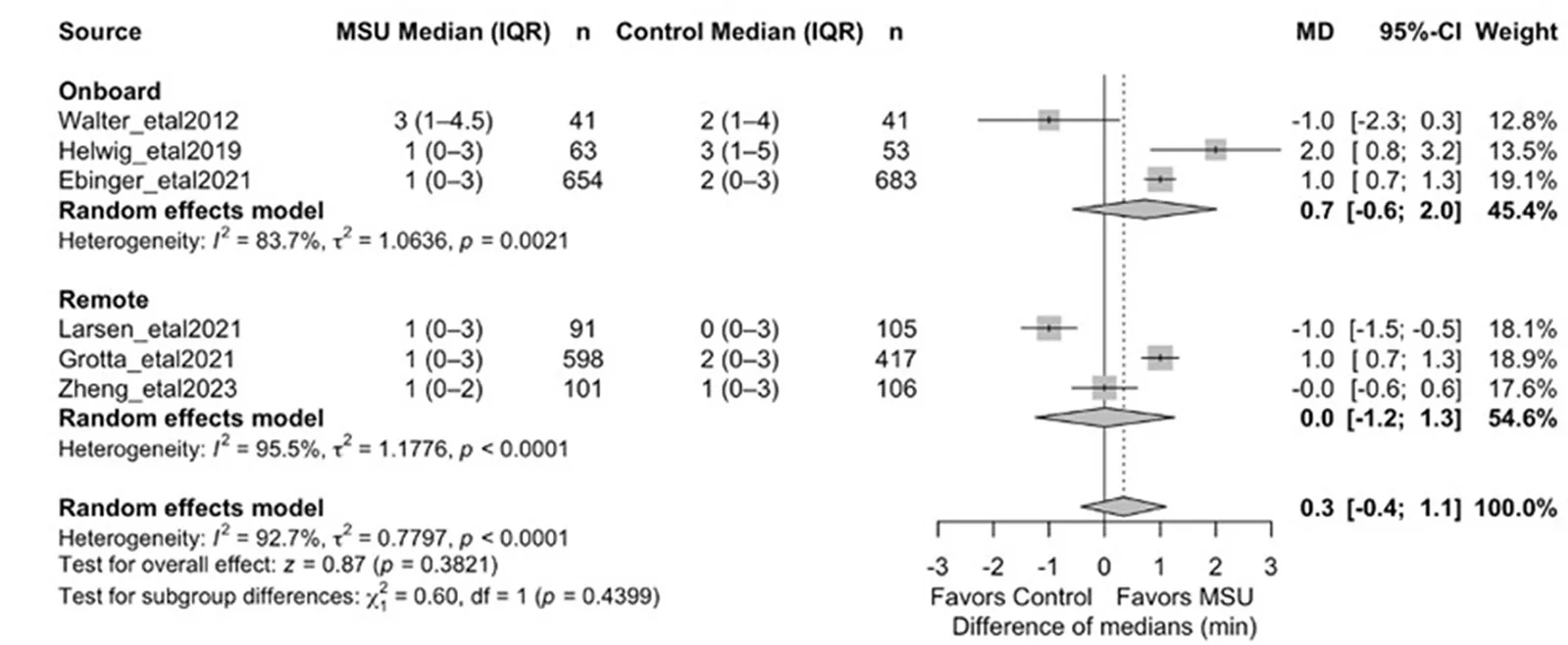
Pooled Medians Difference of Modified Rankin Scale Score Source.

Six studies (*n* = 1,548 MSU; *n* = 1,405 EMS) were included in the analysis to assess the functional outcomes of patients measured by mRS (at 3 months, except Walter et al. (16) at day 7). The pooled median difference of 0.3 [95% CI (-0.4, 1.1), *p* = 0.382] was statistically insignificant. In subgroup analysis, three studies deployed an onboard neurologist and three used a remote neurologist. The onboard neurologist pooled mean difference was 0.7 [95% CI (-0.6, 2.0), *p* = 0.274; *I*^2^= 80%] and the remote neurologist 0.0 [95% CI (-1.2, 1.3), *p*= 0.988; I^2^=100%]. However, subgroup differences were not statistically significant (Figure 3).

#### Safety outcomes

**Figure 4:**
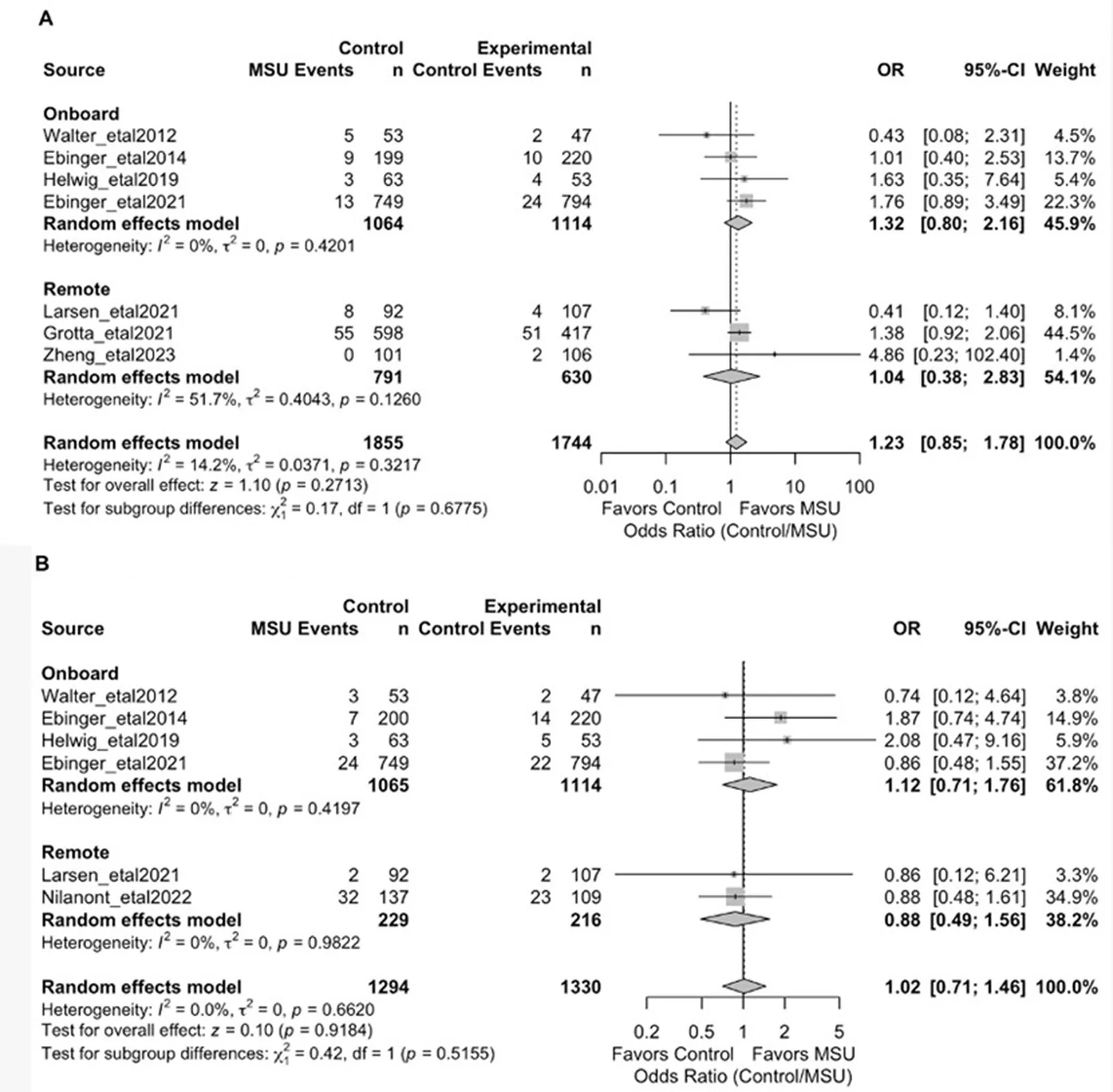
Pooled Odds Ratios of Safety Outcomes. **(A)**: Mortality Events. **(B)**: Intracranial Hemorrhage Events.

Six studies (*n* = 1,294 MSU; *n* = 1,330) were involved in assessing ICH events (*n* = 71 MSU; *n* = 68 EMS), and 7 studies (*n* = 1,855 MSU; *n* = 1,744) were involved in assessing mortality events (*n* = 93 MSU; *n* = 97 EMS). Both safety outcomes showed non-significant pooled odds ratios, ICH 1.019 [95% CI (0.71, 1.45), *p* = 0.918] and mortality 1.23 [95% CI (0.85, 1.77), p = 0.271]. Subgroup analysis by a neurologist configuration (onboard vs. remote) also revealed no statistically significant differences in safety outcomes for ICH (χ^2^ = 0.42, df = 1, *p* = 0.52) or mortality (χ^2^ = 0.17, df = 1, *p* = 0.68). See Figure 4 for details.

## Discussion

This updated systematic review and meta-analysis assessed the impact of MSUs in acute stroke management, focusing on time metrics, patient functionality, and safety outcomes. Across a total of 13,110 patients in four randomized and six non-randomized studies, MSUs have demonstrated consistent, significant reductions in diagnosis and treatment times during transport and after arrival to the hospital, compared with traditional EMS care. Notably, although not statistically significant, there was no increase in mortality or adverse events such as ICH, supporting the aim of MSUs in reducing time metrics without compromising safety outcomes. However, functional outcomes as assessed by mRS scores, did not exhibit significant differences between MSUs and conventional EMS care. To the best of our knowledge, this is also the first review to include subgroup analyses comparing the presence of an onboard versus a remote neurologist in MSUs, providing evidence on their clinical implications.

### Time metrics

Various time metrics are utilized to evaluate the time to different treatment and diagnostic modalities in acute stroke care. This review has found that, across nearly all the essential time metrics investigated, MSUs were significantly faster than standard EMS in providing a diagnosis and treatment. The median reductions in time metrics with MSUs, including alarm-to-thrombolysis time (-26.3 mins), onset-to-thrombolysis time (-35.3 mins), door-to-thrombolysis time (-9.5 mins), door-to-thrombectomy time (-16.5 mins), and time-to-completion of CT (-27.5 mins), were all statistically significant across all studies (*p* < 0.001). Similar reductions were noted almost consistently in the study design and type-of-neurologist subgroups. Only the onset-to-thrombectomy times were not statistically significant, likely reflecting logistical challenges regarding the accurate determination of the time of symptom-onset and hospital-specific protocols after arrival. These pooled estimate findings concur with other meta-analyses in which a mean of ~28–32 min were saved in time-to-treatment and imaging of acute stroke (17–19).

The observed time reductions in this review, ranging from ~10 to 35 min, are clinically important for reperfusion. Given the well-established “save a minute, save a day” and “time is brain” principles in stroke management, in which patients are granted an additional month of a disability-free life for every 15 min reduced from the onset-to-treatment time, any time saved can lead to decreased neuronal loss and improved functional and survival outcomes (2, 20). Additionally, the benefit of intravenous thrombolysis is known to be enhanced with earlier delivery (21). Thus, measures targeting treatment-delaying factors, such as uncertainty about symptom-onset or anti-coagulation status, incorrect triage, uncontrolled blood pressure, treatment before intravenous thrombolysis, and other patient-related demographic and clinical factors, are warranted (22, 23). Although functional outcome differences did not reach statistical significance in the pooled analyses, accelerated time metrics seen with MSUs in this review can potentially provide the known benefits of early reperfusion to this population by mitigating several treatment-delaying factors.

Despite the consistent overall directionality of the reductions in time metrics, the overall high heterogeneity (*I*^2^ = 70–90%) observed in the pooled analyses in this review suggests contextual variability across the studies included. Therefore findings observed across most pooled estimates should be interpreted as directional benefit rather than precise points estimates. This could be attributed to variability in study design, population, and reporting across the included studies represents an important limitation of this review. Although other meta-analyses reported a moderate-to-high heterogeneity, this review included more recent non-RCTs that could have contributed to this variability (18, 19). Nonetheless, time reductions with MSUs persisted irrespective of study design.

### Functional outcomes

Functional outcomes, as measured by mRS, are important assessment tools for the potential long-term clinical effect of MSUs on patients with acute stroke. Yet, across all studies included that had a functional assessment of their participants, no significant differences were found in mRS scores (MD = 0.3, *p* = 0.38) at follow-up across all subgroups. This is consistent with Walter et al. and Zheng et al., who similarly found no significant difference in mRS despite significant time-metric improvements with MSU. Although these findings suggest that reductions in time metrics did not directly reflect improved patient functionality, the absence of statistical significance does not rule out clinical significance, as the small number of studies and sample size prevent the detection of small, but meaningful benefits. There is strong evidence associating quicker reperfusion times with improved functional outcomes that may not appear in aggregate analyses (2, 20). Emberson et al. (21) shows that the benefit of alteplase is proportionally much greater when treatment moves a patient from outside to inside the 4.5-h window, and even more significant when moved to within 3 h (75% increased odds), than when treatment time is merely shortened within the 3–4.5 h window itself (26% increased odds) (21).

Multiple limitations could influence functional outcomes of patients included in this study. The variability across included studies such as differences in follow-up duration, access to rehabilitation after initial treatment, enrollment of mixed ischemic and hemorrhagic stroke, and unmeasured differences in comorbidities and socioeconomic status, can mask a true effect. More importantly, several of the included MSU systems operate within healthcare settings that already have comparatively short baseline EMS times. Therefore, these findings should be interpreted with caution in the presence of current limitations. This highlighted that larger-sized RCTs with standardized functional outcome assessment methods, stratified based on stroke subtype or treatment eligibility, are required to accurately assess the long-term impact of MSUs (14, 15, 24).

### Safety outcomes

It is critical to maintain a safety profile equivalent to that of the standard EMS stroke care when shifting complex medical decisions onto the field via MSUs. This review focused on two major areas regarding stroke care in MSUs: the safety of thrombolytic administration, as demonstrated by the incidence of intracerebral hemorrhage (ICH), and the overall mortality rate. Although not statistically significant, it was found that MSUs had no increase in safety events overall or in any subgroup analyses, as the pooled odds ratio for ICH (MD = 1.019, *p* = 0.918) and mortality (MD = 1.23, *p* = 0.271) did not indicate any safety trade-off. Other meta-analyses and observational studies are consistent with these findings, supporting the safety of pre-hospital neurological assessment, and consequently, earlier thrombolytic administration (19, 25).

It is a major concern that the earlier administration of treatment without proper assessment could lead to more adverse events. However, this review's findings suggest that accelerated workflows outside the hospital did not compromise the safety of patients, especially when combined with diagnostic imaging tools and a physical or virtual neurologist onboard the MSUs. It is crucial to keep in mind that the presence of stroke mimickers in the patient cohort, as well as the variability in workflow, transport times, and in-hospital care readiness, pertaining to differences in regional access and staff experience, could present a challenge in accurately assessing the safety outcomes of MSUs (15, 26). Furthermore, inconsistencies in the timing of mortality measurement (e.g., at discharge, at 90 days, or other follow-up intervals) further complicate the interpretation of safety data across studies (14, 27).

### Remote versus onboard neurologists on MSUs

MSUs may operate under two different configurations, either with an onboard neurologist physically present in the unit or a remote neurologist that performs real-time assessment and imaging interpretations and provides recommendations through a telemedicine system. Understanding how they differ in terms of time metrics, safety profiles, and functional outcomes involved in stroke care can help determine which configuration might be a more cost-effective and sustainable model.

A neurologist model-based subgroup analysis was performed on each of the outcomes of this study using only the included articles in which that respective outcome was reported. Overall, it was found that both onboard and remote neurologists on MSUs significantly decreased treatment time metrics compared to traditional care. However, the magnitude of improvement differed when looking at specific workflow steps. For instance, the reduction was seen more prominently with onboard neurologists in terms of alarm-to-thrombolysis times and time-to-completion of CT, as the data reporting on these time-metrics were sufficient across multiple studies. Although it is not comprehensively reported in the data of the studies included, it is plausible that MSUs with remote neurologists had more impact on post-hospital arrival time metrics and less of an impact on pre-hospital intervals due to communication delays or reliance on the proficiency of the onboard paramedics' or nurses' assessment. Moreover, it can be deduced that the direct assessment and interpretation of the patient by an onboard neurologist accelerates the pre-hospital time metrics. However, studies have shown that remote neurologists are noninferior in terms of workflow performance due to improvement in modern telemedicine systems (28, 29).

There were no differences in safety or functional outcomes in terms of having a remote or an onboard neurologist in the MSUs. In regard to long-term functional improvement, the availability of on-board imaging modalities, earlier treatment administration, and downstream care comprised of reperfusion success and access to rehabilitation could have more of an impact than the type of neurologist model in the MSU. An additional limitation is that the lack of stroke mimic reporting in the included studies compromises the ability to assess triage efficacy across inboard versus remote neurologist configurations, or even MSU versus EMS. Since safety outcomes are similar between the two models, a remote neurologist model is a reasonable and potentially more cost-effective option, freeing up neurologists for other neurological services and simplifying staffing (28). However, additional prospective and randomized studies are needed to further investigate the difference between the effectiveness of these two models.

### Limitations

Several limitations should be acknowledged. Considerable heterogeneity was observed across pooled outcomes (*I*^2^ often 70–90%), likely reflecting variability in study design, patient populations, MSU configurations, outcome reporting, and geographic settings among the included studies. Functional outcome assessment was also inconsistent, with most studies reporting mRS at 3 months while one reported it at day 7. This may limit the generalizability of the conclusions. Finally, data on stroke mimics and cost-effectiveness were limited. Further studies evaluating the cost-effectiveness of MSU implementation are needed to assess the sustainability and scalability of MSU adoption.

## Conclusion

In this review, it was demonstrated that MSUs have markedly improved some of the critical time metrics of acute stroke care through clinically meaningful reductions of ~10–35 min, while maintaining safety outcomes comparable to traditional EMS care. Although functional outcomes did not differ significantly, the improvement in time savings and a stable safety profile supports the effectiveness of MSUs as a modern stroke care system and its broader implementation across different settings. This review also examined the impact of onboard and remote neurologist models in MSUs and found no significant differences, highlighting that remote neurologists in MSUs could potentially be a more cost-effective model. Further, larger randomized controlled studies investigating functional outcomes and formal economic evaluation are warranted to establish the cost effectiveness of MSU deployment.

## Data Availability

The original contributions presented in the study are included in the article/Supplementary material, further inquiries can be directed to the corresponding author.
