# Supplementary material for: Treatment first, transport second: rethinking stroke management with mobile stroke units—A systematic review and meta-analysis of controlled trials
Source: Front Neurol. 2026 Sep 3;17:1852802. doi: 10.3389/fneur.2026.1852802 (PMC13581579; doi:10.3389/fneur.2026.1852802)
Supplement: Supplementary file 1 [file Supplementary_file_1.pdf]

Supplementary Data

Appendix 1. Search strategy & and search results

Appendix 2. Titles excluded during full text screening

Appendix 3. Risk of bias assessment

Appendix 4. publication bias test

Appendix 5. Sensitivity analysis; leave out

Appendix 6. Included studies setting and funding

Appendix 1. Search strategy & and search results

Literature Search:

Databases searched: PubMed, Cochrane, Web of Science, SCOPUS, MEDLINE,

When possible use filter to exclude reviews and non-clinical trials

Export files in RIS extension file please .

**References from Databases**	
**Database and Search Strategy**	**Number of records**
PubMed:<br><br>((("stroke"[MeSH Terms] OR "stroke"[Title/Abstract] OR "acute ischemic stroke"[Title/Abstract] OR "hemorrhagic stroke"[Title/Abstract] OR "transient ischemic attack"[Title/Abstract] OR "TIA"[Title/Abstract])) AND (("mobile stroke unit"[Title/Abstract] OR "MSU"[Title/Abstract]))) AND (("clinical trial"[Publication Type] OR "randomized controlled trial"[Publication Type] OR "randomized"[Title/Abstract] OR "non-randomized"[Title/Abstract]))	**37**
Cochrane Central Register of Controlled Trials (CENTRAL):<br><br>(stroke:ti,ab,kw OR "acute ischemic stroke":ti,ab,kw OR "hemorrhagic stroke":ti,ab,kw OR "transient ischemic attack":ti,ab,kw OR TIA:ti,ab,kw)<br>AND<br>("mobile stroke unit":ti,ab,kw)	68
Web of Science Core Collection:<br><br>TS=(stroke OR "acute ischemic stroke" OR "hemorrhagic stroke" OR "transient ischemic attack" OR TIA)<br>AND<br>TS=("mobile stroke unit" OR MSU)	**63**

SCOPUS:<br><br>TITLE-ABS-KEY(stroke OR "acute ischemic stroke" OR "hemorrhagic stroke" OR "transient ischemic attack" OR TIA)<br>AND<br>TITLE-ABS-KEY("mobile stroke unit" OR MSU)	**174**
MEDLINE:<br><br>1. stroke/ OR brain ischemia/ OR brain infarction/ OR cerebral hemorrhage/ OR transient ischemic attack/<br>2. (stroke OR "acute ischemic stroke" OR "hemorrhagic stroke" OR "transient ischemic attack" OR TIA).ti,ab.<br>3. 1 OR 2<br><br>4. ("mobile stroke unit" OR MSU).ti,ab.<br><br>5. clinical trial.pt. OR controlled clinical trial.pt. OR multicenter study.pt. OR intervention study.pt. OR prospective studies/ OR clinical study.pt. OR clinical studies as topic/<br>OR (clinical trial OR interventional OR prospective OR nonrandomized OR randomized OR non-randomized OR nonrandomised OR randomised OR trial).ti,ab.<br><br>6. 3 AND 4 AND 5	**66**
EMBASE:<br><br>1. stroke/ OR brain ischemia/ OR brain infarction/ OR cerebral hemorrhage/ OR transient ischemic attack/<br>2. (stroke OR "acute ischemic stroke" OR "hemorrhagic stroke" OR "transient ischemic attack" OR TIA).ti,ab.<br>3. 1 OR 2<br><br>4. ("mobile stroke unit" OR MSU).ti,ab.<br><br>5. clinical trial.pt. OR controlled clinical trial.pt. OR random*.ti,ab. OR trial.ti.<br>OR nonrandom*.ti,ab. OR non-random*.ti,ab.<br>OR prospective study/ OR interventional study/ OR experiment subject/<br>OR intervention*.ti,ab. OR prospective.ti,ab.<br><br>6. 3 AND 4 AND 5	**Non aplicaple**
**References from :(for other gray sources or crossref)**	

Method used (ex. Crossref , or registry)	Number of records
[Clinicaltrail.gov]<br><br>**Condition:** ischemic stroke OR cerebral infarction OR brain ischemia<br><br>**Intervention:** mobile stroke unit	10
**crossreferencing**	**1 (PHANTOM-S study)**
**Conclusion**	
**Total Number of Research Items**	**408 (databases only)<br>10 (Registries)<br>1 (crossref)<br>419(total)**
**Duplicates Removed**	**184**
**Unique Items for Phase I Screening(will be screened)**	**235**
**Items removed during phase I screening**	**209**
**Items Sought for Phase II Screening(result of Phase1)**	**26**
**Items Screened During Phase II Screening**<br>(note: sometimes this is different from the items sought because some items may not be located)	**26**
**Final excluded records**	**#16**
**Exclusion Reason 1**	**See PRISMA figure**
**Exclusion Reason 2**	**See PRISMA figure**
**Final included records in SR**	**#10**
**Final included records in MA**	**#10**
**Exclusion from MS Reason 1**	**-**
**Exclusion from MS Reason 2**	**-**

Appendix 2. Table 1. Titles excluded during full text screening

Appendix 2. Titles excluded during full text screening

Name of the study	Location	Design
Jessica L. et al. (2023)	Effect of Mobile Stroke Unit Dispatch in all Patients with Acute Stroke or TIA patients with dysphagia	Post-hoc analysis
Bowry, et al. (2018)	Time to Decision and Treatment With tPA (Tissue-Type Plasminogen Activator) Using Telemedicine Versus an Onboard Neurologist on a Mobile Stroke Unit	Wrong control.
Gangfeng Gu, et al. (2021)	Building a Mobile Stroke Unit Based on 5G Technology – A Study Protocol	Protocol
Bowry, et al. (2015)	Benefits of Stroke Treatment Using a Mobile Stroke Unit Compared With Standard Management The BEST-MSU Study Run-In Phase	Run-in-phase of BEST-MSU study
Xuya Huang, et al. (2015)	Alteplase versus tenecteplase for thrombolysis after ischaemic stroke (ATTEST): a phase 2, randomised, open-label, blinded endpoint study	Wrong intervention, hospital based intervention

Gurpreet Chaggar1, et al. (2025)	Impact of Clinical Trial Enrollment on Thrombolysis Workflow in a Mobile Stroke Unit: Results from the AcT Trial	Post-hoc analysis
Yogendr kumar, et al. (2024)	Evaluating the Safety and Efficacy of Telemedicine Physician Assessments on a Mobile Stroke Unit: Protocol for a Prospective Open-Label Blinded End-Point Randomized Controlled Trial	Protocol
Jose-Migel YamalI, et al. (2020)	Successful conduct of an acute stroke clinical trial during COVID	Post-hoc analysis
Peter M, et al. (2016)	Influence of Distance to Scene on Time to Thrombolysis in a Specialized Stroke Ambulance	Post-hoc analysis
Jason Mackey, et al. (2023)	Golden Hour Treatment With tPA (Tissue-Type Plasminogen Activator) in the BEST-MSU Study	Post-hoc analysis
Czap, et al. (2020)	Mobile Stroke Unit Computed Tomography Angiography Substantially Shortens Door-to-Puncture Time	Post-hoc analysis
Ebinger1, et al. (2012)	PHANTOM-S: the prehospital acute neurological therapy and optimization of medical care in stroke patients – study	protocol

Yamal1, et al. (2018)	Benefits of stroke treatment delivered using a mobile stroke unit trial	Protocol
Suja S, at el (2024)	A Prospective Multicenter Analysis of Mobile Stroke Unit Cost-Effectiveness	Economic study
Grotta, at el (2021)	Mobile stroke units can improve stroke outcomes	This is a brief insight of a duplicate article. NOVEMBER 2021 | volume 17. www.nature.com/nrneuro
Navi, et al. (2023)	Strokes Averted by Intravenous Thrombolysis: A Secondary Analysis of a Prospective, Multicenter, Controlled Trial of Mobile Stroke Units	Post-hoc analysis

Appendix 3. Risk of bias assessment

3.1 ROB2 Risk of bias assessment tool

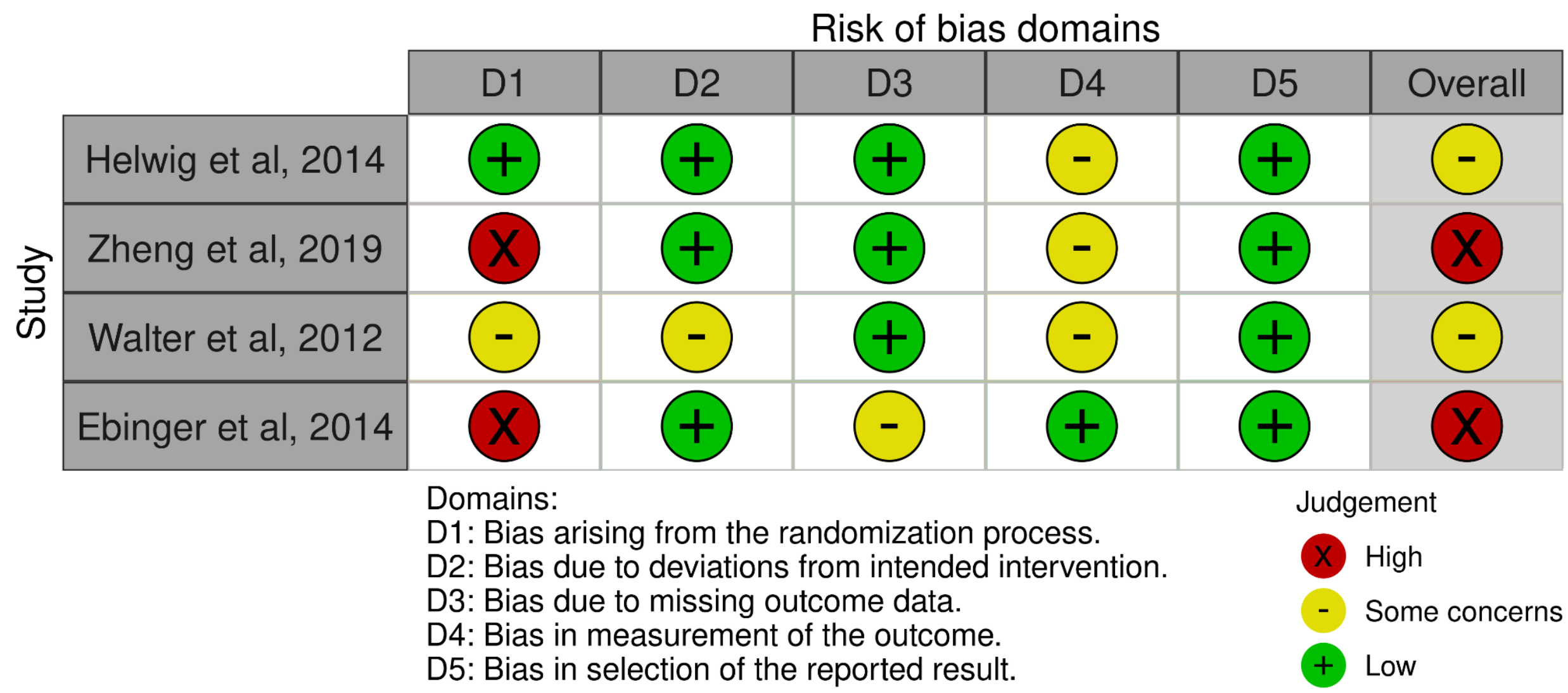

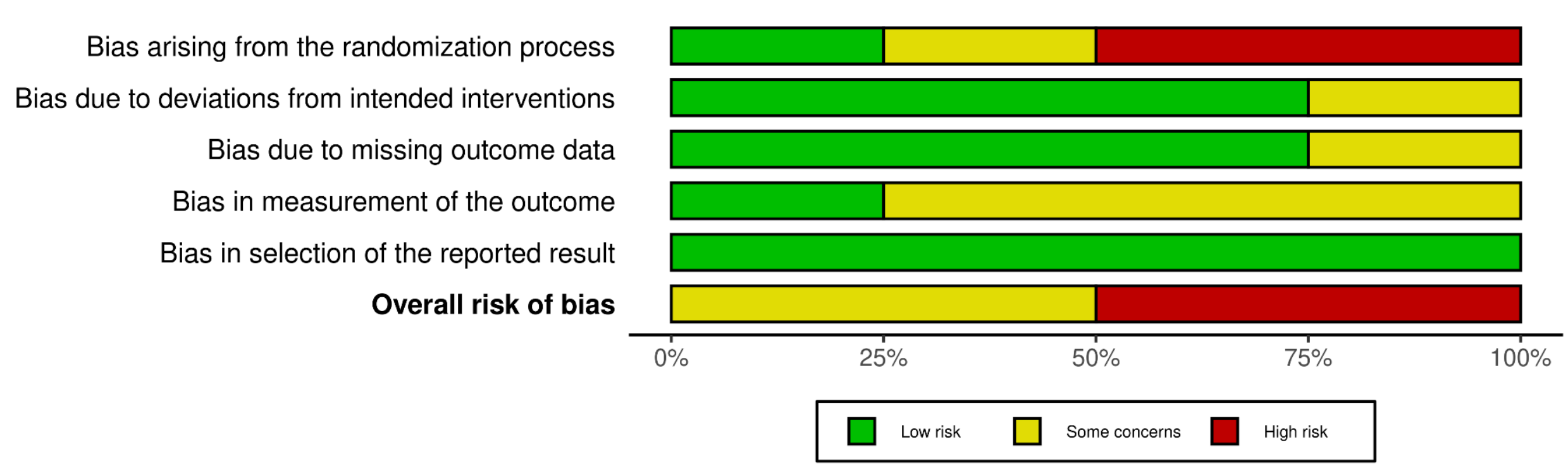

3.2 ROBINS-I Risk of bias assessment tool

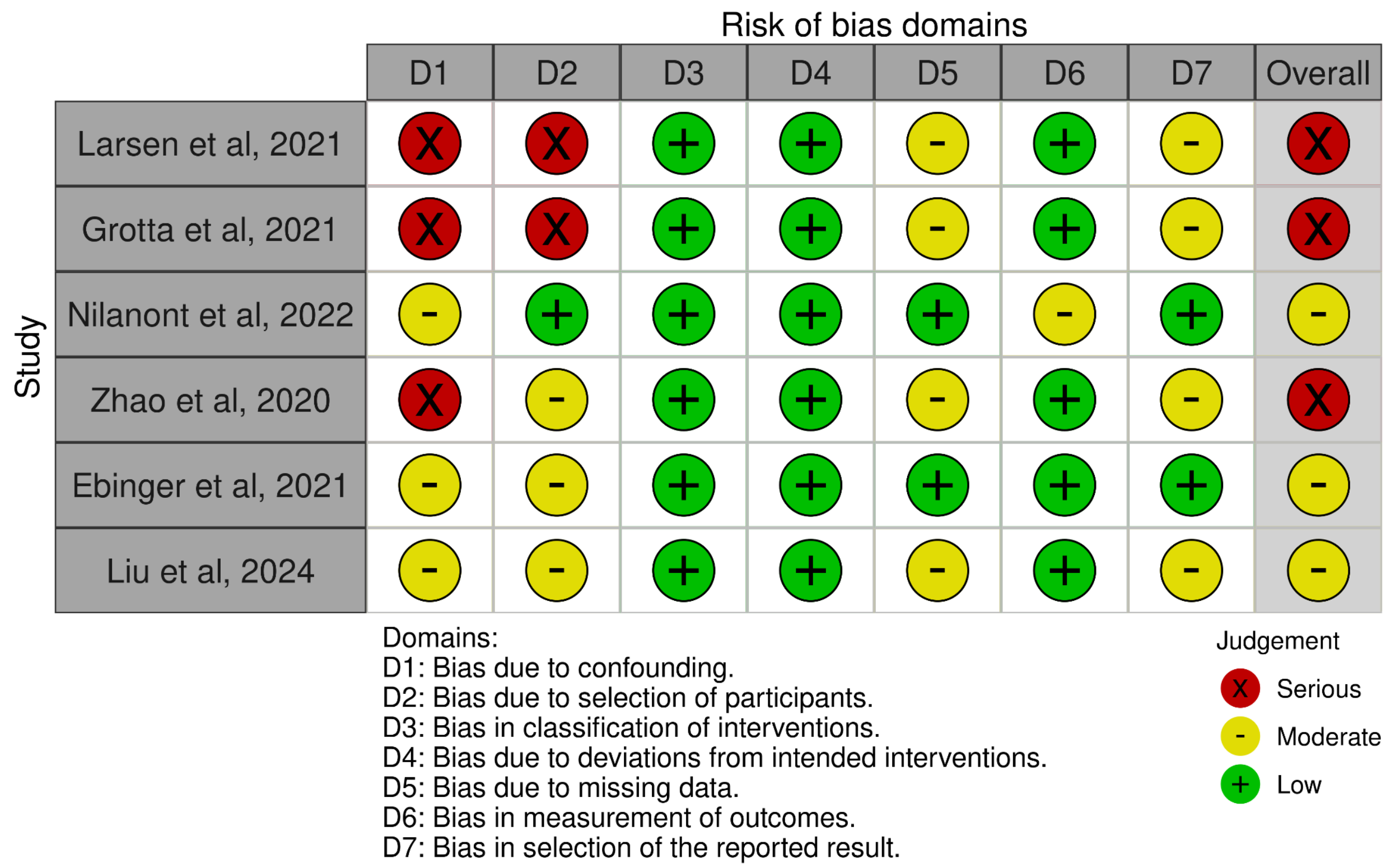

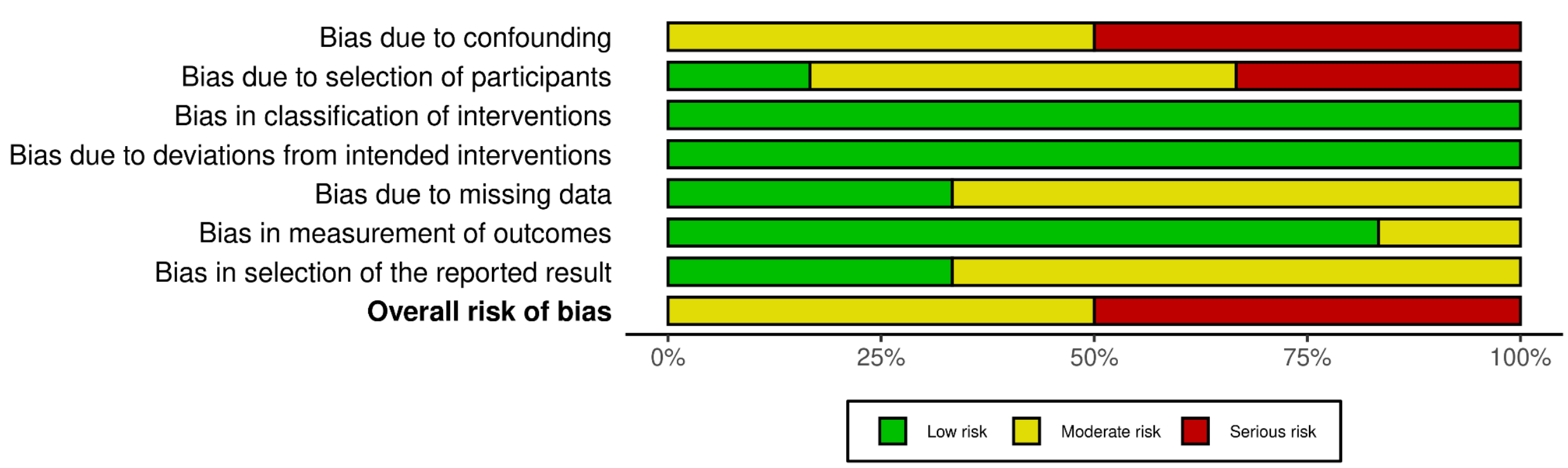

Appendix 4. Publication bias tests

4.1 Alarm to Thrombolysis Time

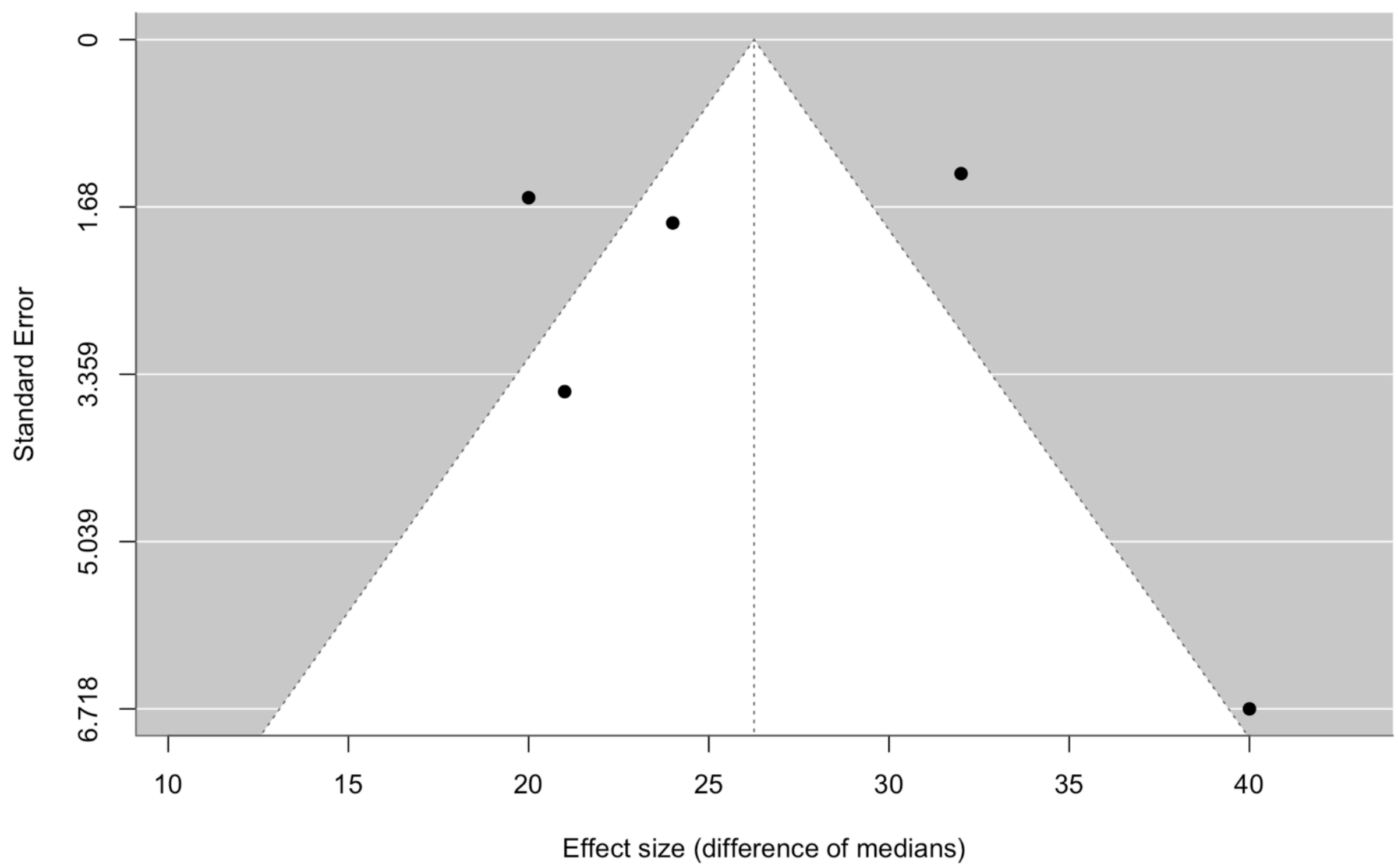

4.2 Onset to Thrombolysis Time

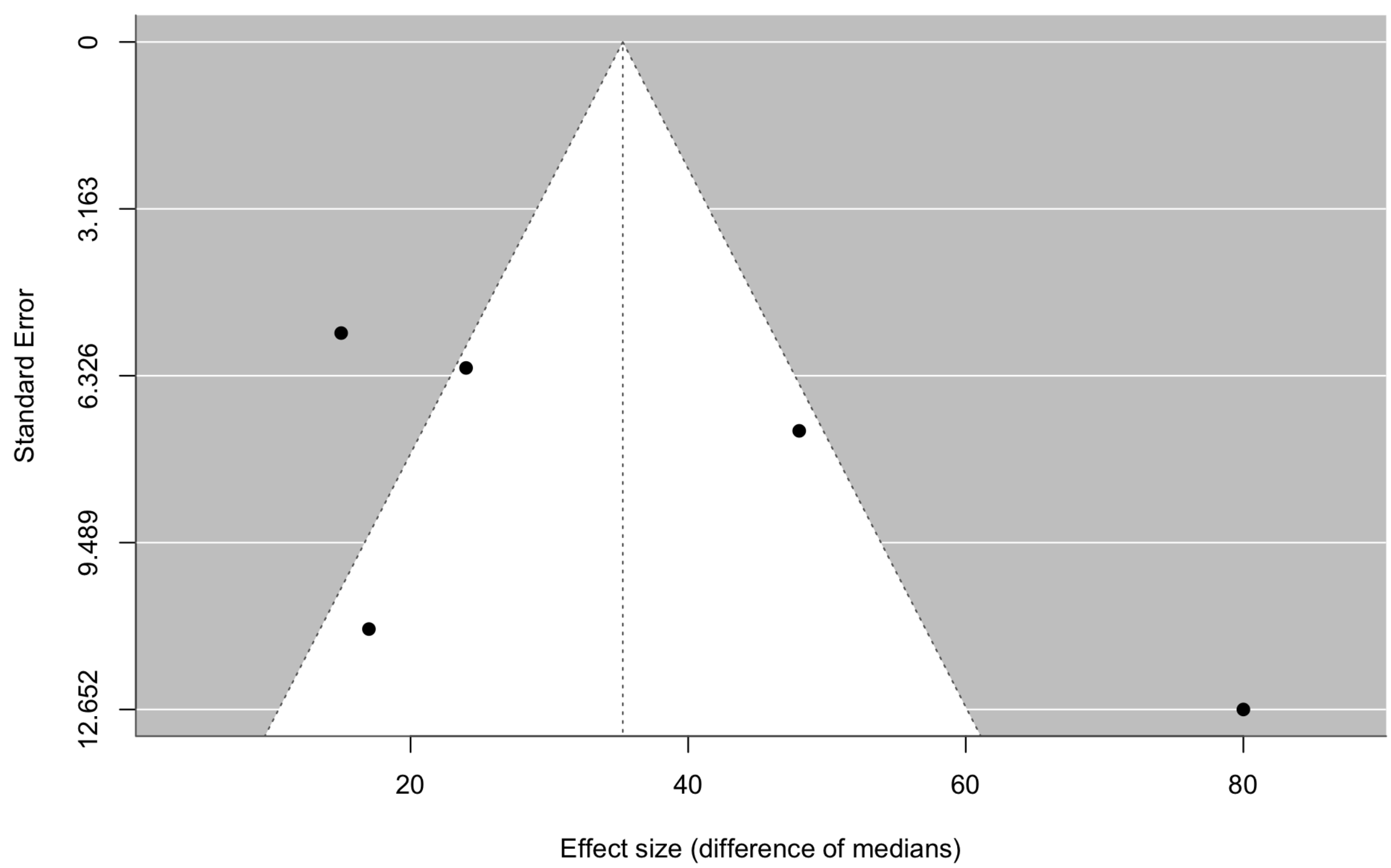

4.3 Door Thrombolysis Time

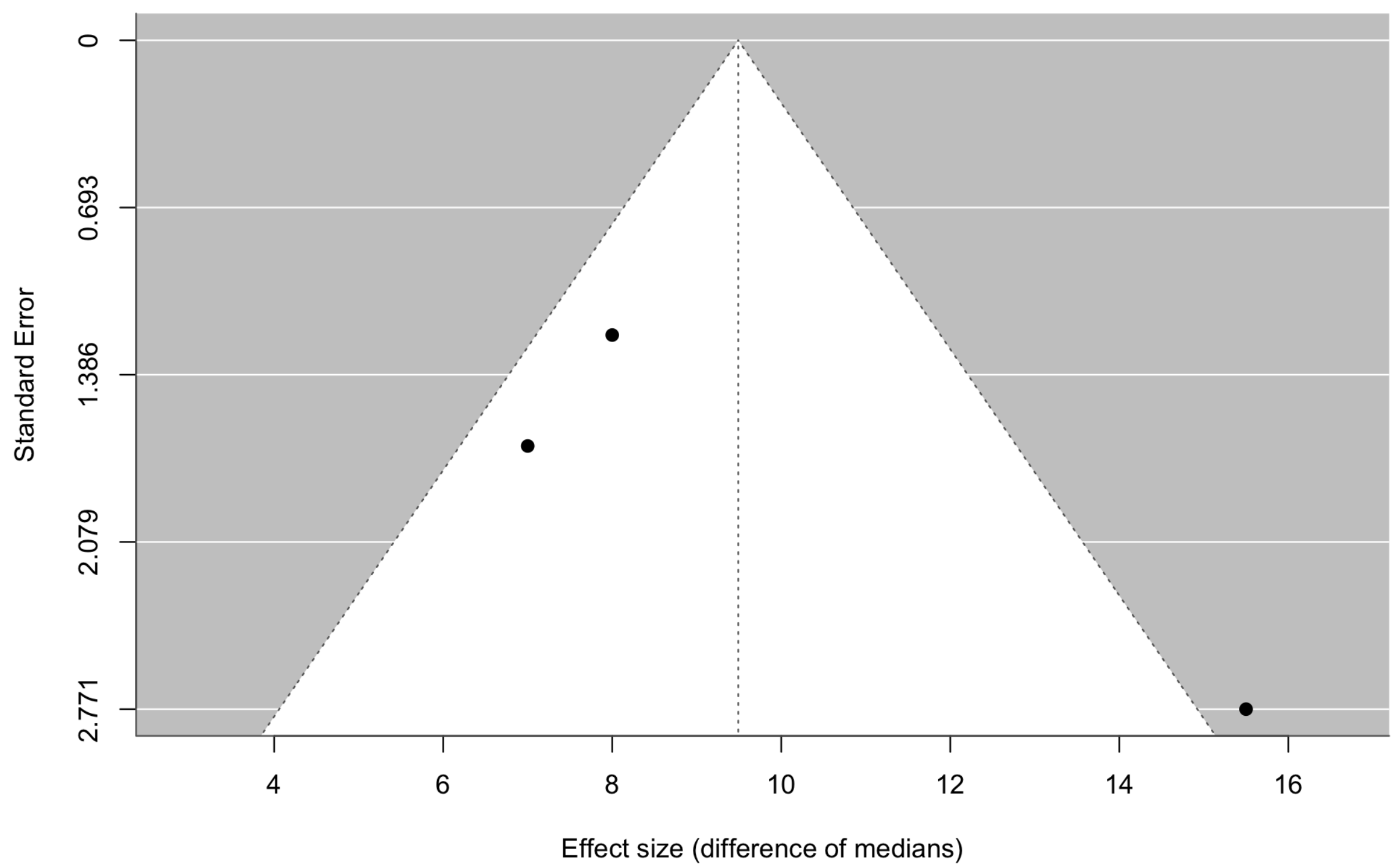

4.4 Door Thromboectomy Time

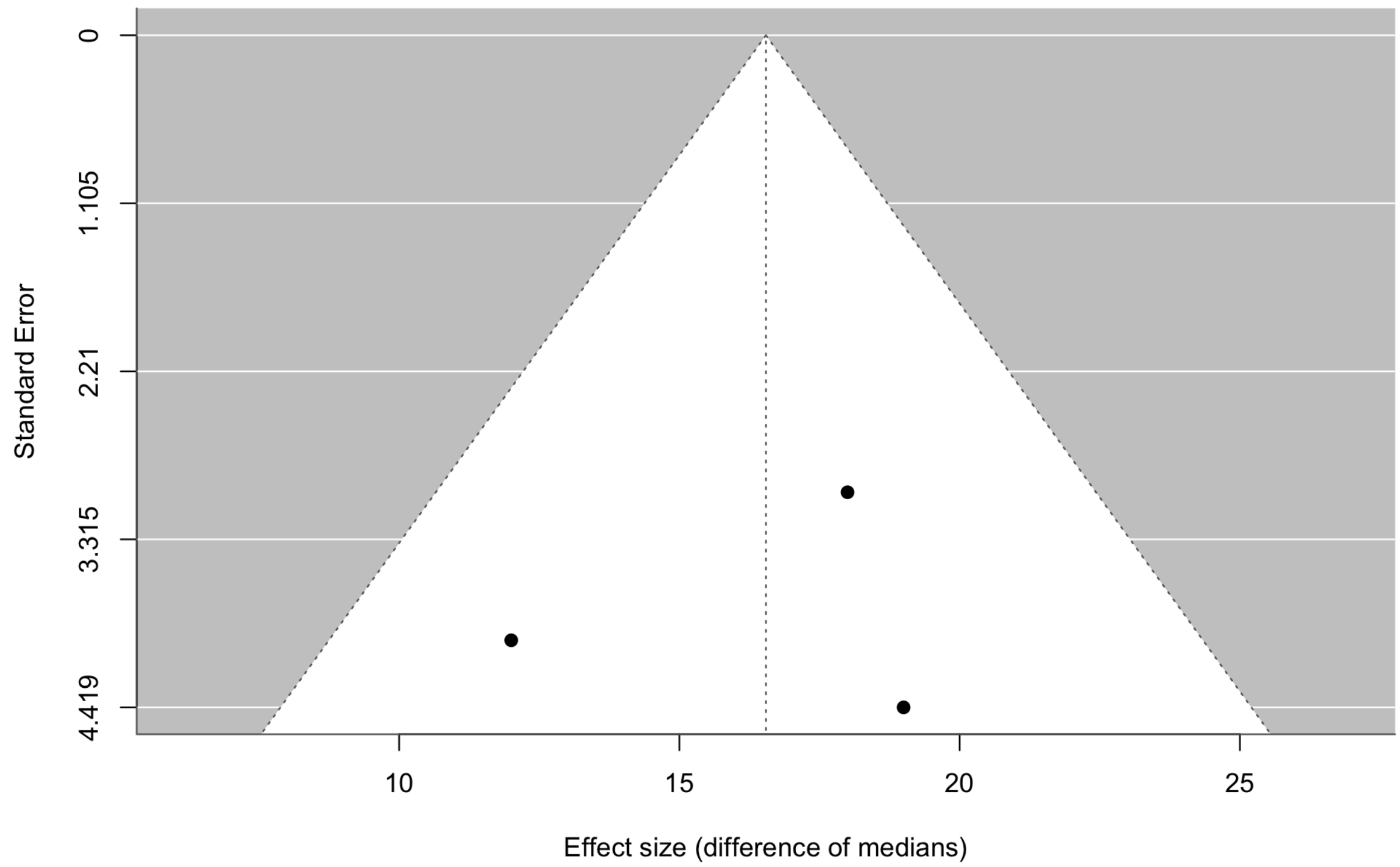

4.5 Time to end of CT

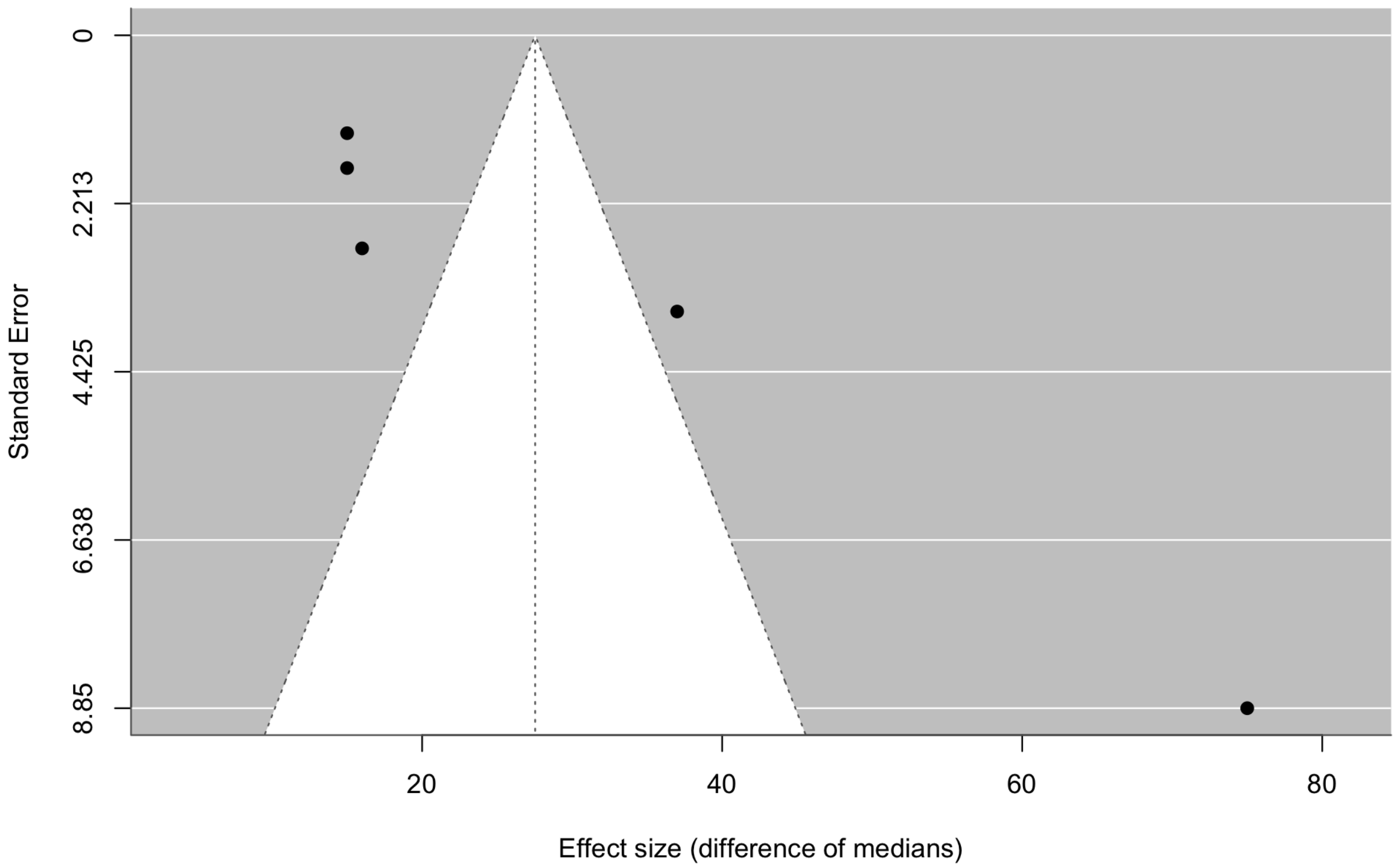

4.6 functional outcome, MRS score

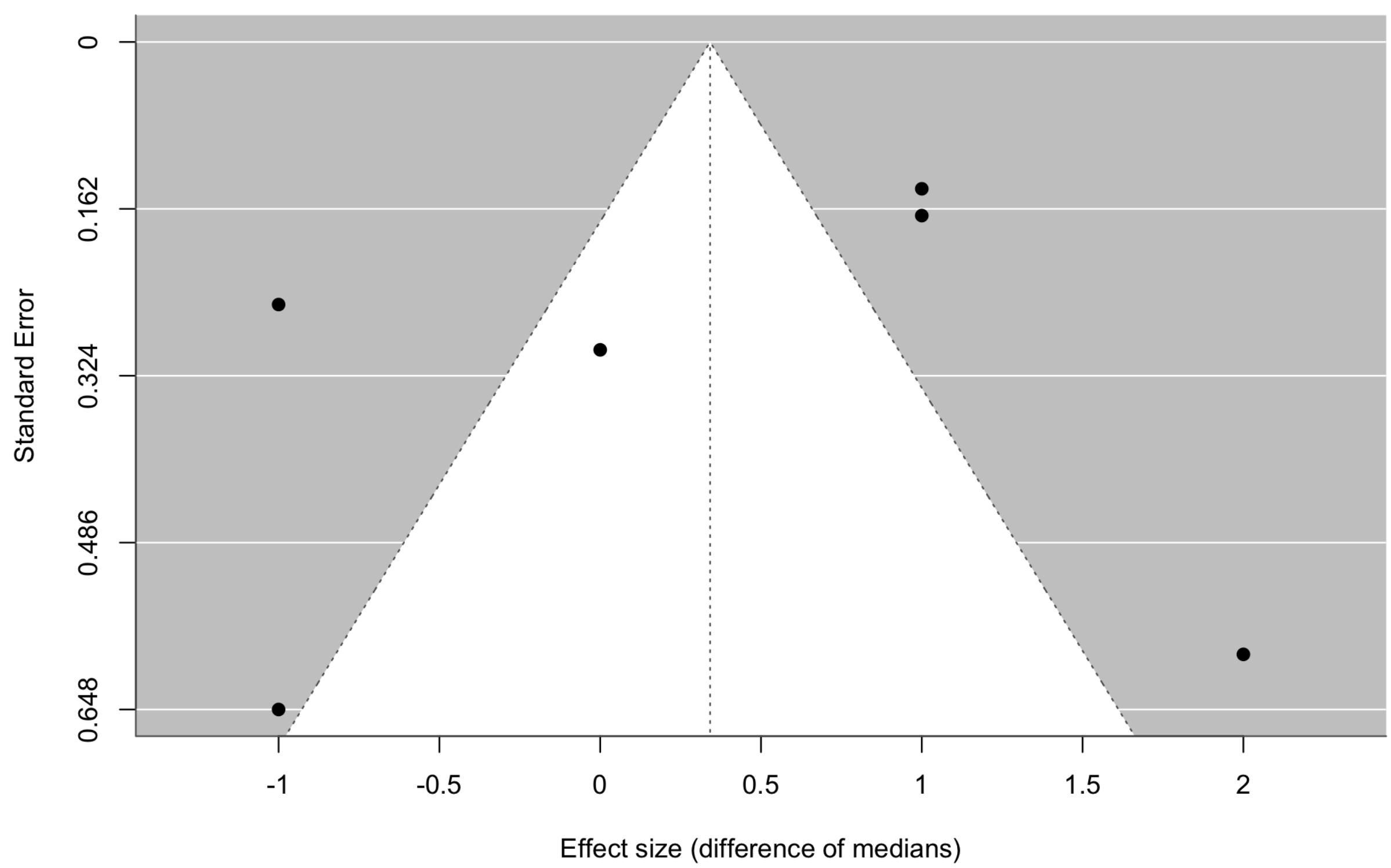

4.7 Safety Outcome, Mortality

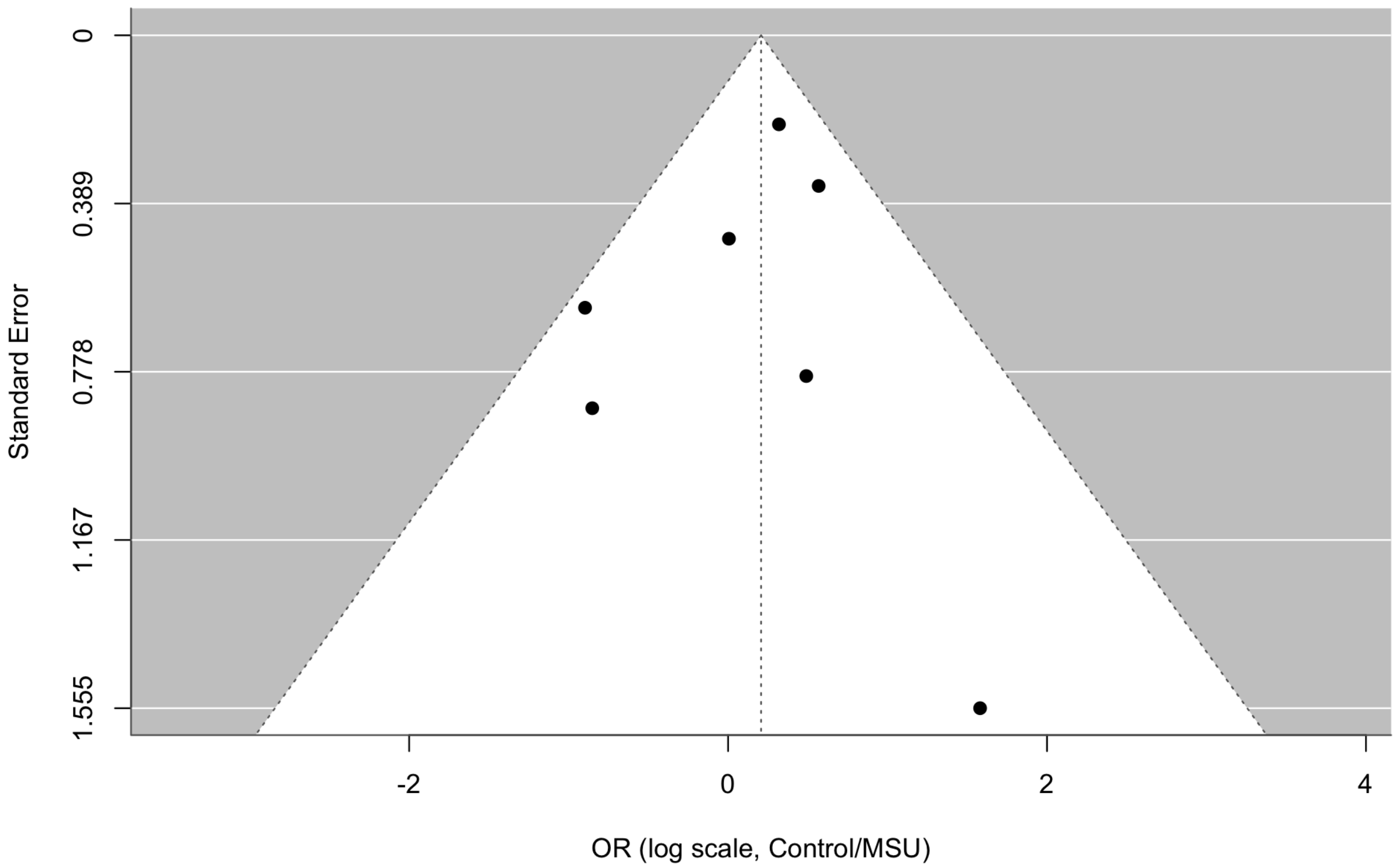

4.8 Safety Outcome; Intracranial Hemorrhage

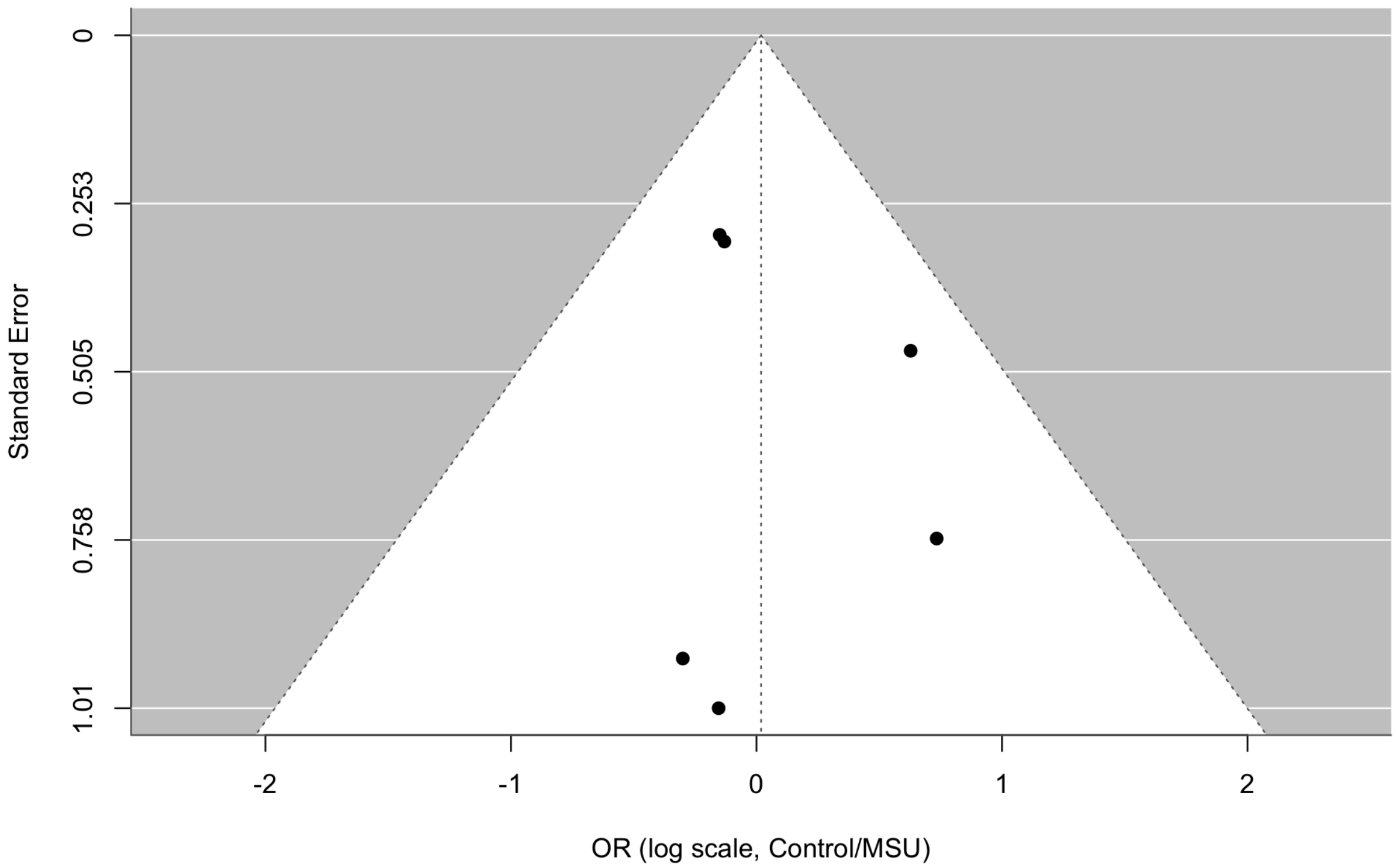

Appendix 5. Sensitivity analysis; leave out

5.1 Alarm to Thrombolysis Time

Study	Pooled difference (min)	se	pval	ci.lb	ci.ub	tau2	I2
Walter_et al2012	24.46	3.27	6.864	18.06	30.86	37.98	91.97
Ebinger_etal2014	27.19	4.27	1.9881	18.81	35.56	60.91	92.48
Ebinger_etal2021	28.04	3.29	1.4458	21.60	34.48	32.31	85.08
Larsen_et al2021	27.54	3.62	3.0284	20.43	34.64	43.70	92.38
Grotta_et al2021	23.55	2.45	6.296	18.75	28.34	14.68	70.03

5.2 Onset to Thrombolysis Time

Study	Pooled difference (min)	se	pval	ci.lb	ci.ub	tau2	I2
Walter_et al2012	26.17	7.62	0.0006	11.23	41.10	175.59	77.88
Ebinger_etal2014	38.93	13.54	0.0040	12.40	65.47	644.47	90.10
Zhao_etal 2020	41.18	11.76	0.0005	18.14	64.22	463.24	85.99
Ebinger_etal2021	31.96	11.09	0.0040	10.21	53.70	410.27	86.75
Larsen_et al2021	39.61	11.50	0.0006	17.07	62.16	463.29	89.97

5.3 Door Thrombolysis Time

Study	Pooled difference (min)	se	pval	ci.lb	ci.ub	tau2	I2
Zhao_etal 2020	7.65	0.99	0.0000	5.72	9.59	0.00	0.00
Larsen_et al2021	11.34	3.73	0.0024	4.03	18.64	23.54	83.69
Nilanont_ etal2022	10.96	4.24	0.0097	2.65	19.28	30.87	85.46
Zhao_etal 2020	7.65	0.99	0.0000	5.72	9.59	0.00	0.00
Larsen_et al2021	11.34	3.73	0.0024	4.03	18.64	23.54	83.69

5.4 Door Thromboectomy Time

Study	Pooled difference (min)	se	pval	ci.lb	ci.ub	tau2	I2
Zhao_etal 2020	15.57	2.95	0.000	9.79	21.34	5.57	30.96
Grotta_et al2021	15.24	3.49	0.000	8.40	22.08	6.82	27.84
Nilanont_ etal2022	18.32	2.48	0.000	13.45	23.19	0.00	0.00
Zhao_etal 2020	15.57	2.95	0.000	9.79	21.34	5.57	30.96
Grotta_et al2021	15.24	3.49	0.000	8.40	22.08	6.82	27.84

5.5 Time to end of CT

Study	Pooled difference (min)	se	pval	ci.lb	ci.ub	tau2	I2
Walter_et al2012	23.77	4.56	0.0000	14.83	32.72	68.74	93.38
Ebinger_et al2014	33.18	7.81	0.0000	17.87	48.48	221.71	95.76
Ebinger_et al2021	32.99	7.55	0.0000	18.20	47.79	206.29	95.99
Larsen_et al2021	31.50	6.18	0.0000	19.40	43.61	133.82	96.10
Zheng_et al2023	20.06	3.55	0.0000	13.11	27.01	44.27	91.17

4.6 functional outcome, MRS score

Study	Pooled difference (min)	se	pval	ci.lb	ci.ub	tau2	I2
Walter_et al2012	0.54	0.41	0.1904	-0.27	1.34	0.74	93.56
Helwig_et al2019	0.08	0.42	0.8381	-0.73	0.90	0.76	93.66
Ebinger_et al2021	0.19	0.53	0.7180	-0.84	1.23	1.22	92.90
Larsen_et al2021	0.68	0.29	0.0584	0.11	1.24	0.28	81.69
Grotta_et al2021	0.19	0.53	0.7173	-0.85	1.23	1.23	93.48

5.7 Safety Outcome

Study	Pooled difference (min)	se	pval	ci.lb	ci.ub	tau2	I2
Walter_et al2012	0.03	0.19	0.8662	-0.33	0.40	0.00	0.00
Ebinger_et al2014	-0.09	0.20	0.6571	-0.48	0.30	0.00	0.00
Helwig_et al2019	-0.03	0.19	0.8913	-0.40	0.34	0.00	0.00
Ebinger_et al2021	0.12	0.23	0.6068	-0.33	0.57	0.00	0.00
Larsen_et al2021	0.02	0.19	0.8947	-0.34	0.39	0.00	0.00

Appendix 6. Table 1. Included studies setting and funding

Appendix 6. Included studies setting and funding

Name of the study	Funding	setting
Helwig et al. (2014)	Funded by the Ministry of Health of the Saarland.	Nonurban
Zheng et al. (2023)	Funded by Sichuan Science and Technology Department Project, YaanScience and Technology Department Project, Central Government Guides Local Scientific and Technological Development Project, Innovation Research Project, and Sichuan Provincial Health Commission Special Clinical Research Project,	Mixed urban and nonurban
Larsen et al. (2021)	Funded by the Norwegian Air Ambulance Foundation, a non-profit organization.	Nonurban
Grotta et al. (2021)	Funded by the Patient-Centered Outcomes Research Institute. Also, Genentech supplied t-PA/Activase free of charge and Chiesi USA supplied Cardene I.V. free of charge.	Urban
Nilanont et al. (2023)	Funded by the Thaicom and the Siriraj Foundations.	urban

Walter et al. (2012)	Funded by Ministry of Health of the Saarland, Germany, the Werner-Jackstädt Foundation, the Else-Kröner-Fresenius Foundation, and the Rettungsstiftung Saar.	Mixed urban and nonurban
Zhao et al. (2020)	Funded by the Australian Commonwealth Government, Victorian State Government, Royal Melbourne Hospital Neurosciences Foundation, Stroke Foundation, The Florey Institute of Neurosciences and Mental Health, the University of Melbourne, Boehringer Ingelheim, and private donation.	Urban
Ebinger et al. (2021) 21	Funded by the Deutsche Forschungs gemeinschaft(DFG, German Research Foundation), the B-SPATIAL registry, by the Federal Ministry of Education and Research via the Center for Stroke Research Berlin. Additional analyses requested during the review process and performed by the Institut für Biometrie und klinische Epidemiologie, Charité Universitätsmedizin Berlin was funded by the Verein zur Förderung der Hirnforschung und Rehabilitation e.V.	Urban
Liu et al. (2024)	Funded by the Key Project of Natural Science Research in Anhui Universities	Nonurban
Ebinger et al. (2014) 23	Funded by the Zukunftsfonds Berlin and the Technology Foundation Berlin with EU co-financing by the European Regional Development Fund (ERDF)	Urban
